# Identification of clinically significant psychological distress and psychiatric morbidity by examining quality of life in subjects with occupational asthma

**DOI:** 10.1186/1477-7525-9-76

**Published:** 2011-09-22

**Authors:** David Miedinger, Kim L Lavoie, Jocelyne L'Archeveque, Heberto Ghezzo, Jean-Luc Malo

**Affiliations:** 1 Division of Chest Medicine, Research Center, Department of Chest Medicine, Hôpital du Sacré-Cœur de Montréal - a University of Montreal affiliated hospital, 5400 Gouin West, Montréal, Québec, H4J 1C5, Canada; 2 Department of Psychology, University of Quebec at Montreal (UQAM), P.O. Box 8888, Succursale Center-Ville, Montreal, Quebec, H3C 3P8, Canada; 3 Montreal Behavioural Medicine Centre, Research Center, Montreal Heart Institute - a University of Montreal affiliated hospital, 5000 Belanger, Montreal, Quebec, H1T 1C8, Canada

**Keywords:** Occupational asthma, psychiatric disorder, psychological distress, screening, quality of life

## Abstract

**Background:**

The Juniper Asthma Specific Quality of Life Questionnaire (AQLQ(S)) is a questionnaire that allows measurement of disease specific quality of life. We wanted to examine correlations between the (AQLQ(S)) general and different subscale scores and both psychiatric morbidity and levels of psychological distress in individuals with occupational asthma (OA) and to determine if results in the emotional function subscale allow identification of individuals with clinically significant psychological distress or current psychiatric disorders.

**Methods:**

This was a cross-sectional study of individuals with OA who were assessed during a re-evaluation for permanent disability, after they were no longer exposed to the sensitizing agent. Patients underwent a general sociodemographic and medical history evaluation, a brief psychiatric interview (Primary Care Evaluation of Mental Disorders, PRIME-MD) and completed a battery of questionnaires including the AQLQ(S), the St-Georges Respiratory Questionnaire (SGRQ), and the Psychiatric Symptom Index (PSI).

**Results:**

There was good internal consistency (Cronbach alpha = 0.936 for the AQLQ(S) total score) and construct validity for the AQLQ(S) (Spearman rho = -0.693 for the SGRQ symptom score and rho = -0.650 for the asthma severity score). There were medium to large correlations between the total score of the AQLQ(S) and the SGRQ symptom score (r = -.693), and PSI total (r = -.619) and subscale scores (including depression, r = -.419; anxiety, r = -.664; anger, r = -.367; cognitive disturbances, r = -.419). A cut-off of 5.1 on the AQLQ(S) emotional function subscale (where 0 = high impairment and 7 = no impairment) had the best discriminative value to distinguish individuals with or without clinically significant psychiatric distress according to the PSI, and a cut-off of 4.7 best distinguished individuals with or without a current psychiatric disorder according to the PRIME-MD.

**Conclusions:**

Impaired quality of life is associated with psychological distress and psychiatric disorders in individuals with OA. Findings suggest that the AQLQ(S) questionnaire may be used to identify patients with potentially clinically significant levels of psychological distress.

## Background

Asthma is a chronic inflammatory disorder of the airways. Occupational asthma (OA) is asthma that is caused and maintained by conditions attributable to the occupational environment and not to stimuli encountered outside the workplace [1]. The impact of a disease on a patient's health and well-being is individual. According to Paul Jones, "A patient's health-related quality of life is the result of a generic disturbance to health common to all patients with the disease, modulated by factors that are internal and unique to the individual." [2]. Health-related quality of life questionnaires should therefore contain items evaluating physical, psychological and social domains, and in general, the item content of a questionnaire should be derived from patients rather than health professionals [3].

There are a variety of different measures available to determine asthma-related quality of life according to a recent review [4]. One of the most commonly used measures is the Asthma Quality of Life Questionnaire (AQLQ) developed in Canada by Juniper and co-workers. This questionnaire is available in approximately 80 languages, and changes in AQLQ scores have been shown to have strong correlations with changes in asthma control and medication usage [5]. Juniper and co-workers found moderate cross-sectional correlations of the AQLQ subscales with the psychosocial function domain of the Sickness Impact Profile and the emotion subscale of the Rand General Health Survey [5]. The standardized version of the AQLQ(S) has been shown to have a moderate cross-sectional correlation (r = 0.48) with the mental component summary measures of the Short Form-36 questionnaire [5,6].

Previous research has demonstrated a link between health-related quality of life and an increased risk of all-cause mortality and healthcare use in individuals with asthma [7,8]. The goal of asthma treatment is therefore to gain control of symptoms, which relies upon various self-management behaviors such as daily symptom self-monitoring, adherence to medication, refraining from smoking, as well as managing environmental asthma triggers. Chronic negative mood states such as depressive or anxiety disorders may interfere with motivation to engage in these self-management behaviors, and have been linked to worse asthma-related quality of life [9]. Having depression has also been shown to be associated with medication non-adherence in patients suffering from different medical conditions,[10] and anxiety disorders have been shown to be related to increased use of bronchodilators (reliever medication) and decreased use of controller medication such as inhaled corticosteroids among asthmatics [9,11]. Early diagnosis of chronic negative mood states therefore offers the opportunity to begin specific anti-anxiety or anti-depressive therapy (i.e., psychotherapy or pharmacotherapy), and has the potential to reverse these adverse disease outcomes.

The aim of this study was to assess the correlation between asthma-specific quality of life and both levels of psychological distress and psychiatric disorders assessed by standardized tools, in patients with OA. Specifically, this study examined the correlation between general and different subscale scores on the AQLQ(S) on the one hand and, on the other hand, levels of psychological distress and rates of psychiatric disorders, and the extent to which the responses on the emotion subscale of the AQLQ(S) allowed for the identification of individuals with significant psychological distress or psychiatric disorders (i.e., patients who met diagnostic criteria for depressive and anxiety disorders according to the Diagnostic and Statistical Manual of Mental Disorders, 4^th ^Edition, DSM-IV).

## Methods

### Study design, setting and participants

This was a cross-sectional study of patients who claimed compensation for OA at the Workers' Compensation Agency of Quebec (Commission de la santé et sécurité du travail du Québec; CSST) in the years 2004 to 2006. Patients who were no longer exposed to the sensitizing agents causing OA for two years or more were evaluated by two of the four Quebec CSST medical committees in Montreal (Montreal Chest Institute and Hôpital du Sacré-Coeur) for a permanent disability indemnity. In Quebec, all patients who claim compensation for OA undergo specific inhalation testing to confirm a diagnosis of OA. All claimants scheduled for evaluation by the committees were asked to participate in this study on a voluntary basis. Patients were assessed when the participants were re-evaluated to determine compensation for permanent disability. Patients were assured that the medical committee would not be informed of participation, nor of the assessment results. They were given compensation for their study participation to cover expenses like loss of salary and transportation or parking fees. All study participants gave written, informed consent for their participation. The research protocol was approved by the Research Ethical Committee of Hôpital du Sacré-Coeur de Montreal.

### Measures

All patients underwent standard spirometry and methacholine challenge testing. All patients completed a questionnaire on chest and upper airway symptoms, medication use, home allergen exposure, and smoking status. Patients also completed a questionnaire assessing whether they were still exposed to the sensitizing agent, as well as a questionnaire assessing socio-economic factors. In addition, participants completed the validated French versions of the following questionnaires:

### Asthma Quality of Life Questionnaire (AQLQ(S))

The standardized version of the AQLQ(S) includes 32 items and evaluates asthma quality of life across four domains that may be negatively affected by asthma. The domains include 1) asthma symptoms 2) activity limitations 3) emotional function and 4) exposure to environmental stimuli. Every question is scored from one (severe impairment) to seven (no impairment), and the total score is the mean of the four scores [6]. Information on psychometric properties of this instrument have been published [12].

### St-Georges Respiratory Questionnaire (SGRQ)

For this study, patients completed the section on respiratory symptoms from the SGRQ to assess the patient's perception of their recent respiratory problems.

This section of the questionnaire includes eight items where individuals choose the appropriate answer on a 5-point Likert scale. Each item in the questionnaire has an empirically derived weight. The total score is representing the sum of these items. The total score ranges from 0 to 100 and a higher score indicates a worse symptom-related HRQoL [13]. Information on psychometric properties of this instrument have been published [12].

### Psychiatric Symptom Index (PSI)

The PSI is a 29-item questionnaire designed to assess the presence and intensity of psychological distress levels in the past two weeks [14]. Items are scored using a four-point scale from 0 (never) to 3 (very often). Total and subscale scores (depression, anxiety, anger and cognitive disturbance) are calculated as a percentage of the total possible score out of 100. Scores of > 20 are considered to indicate clinically significant levels of psychological distress [14]. Information on psychometric properties of this instrument have been published [15].

### Primary Care Evaluation of Mental Disorders (Prime-MD)

The PRIME-MD is a validated brief screening instrument designed to detect some of the most common psychiatric disorders seen in community and medical settings [16]. It consists of a 27-item patient self-report section followed by a structured clinical interview that is used to follow-up patient responses. The PRIME-MD evaluates 5 groups of mental disorders (mood, anxiety, somatoform, alcohol, eating), and items are based on the diagnostic criteria from the DSM [17]. It has demonstrated very good sensitivity (83%) for any psychiatric diagnosis and excellent specificity (88%) across diagnostic modules [16]. We administered the mood (depressive) and anxiety disorder modules, given they are the most prevalent psychiatric disorders seen in asthma patients. We then classified individuals in two groups: either as having a mood and/or anxiety disorder (*any psychiatric disorder*) or not having either mood or anxiety disorder (*no psychiatric disorder*). Information on psychometric properties of this instrument have been published [18].

### Spirometry and Methacholine Provocation Testing

All patients underwent standard spirometry according to ATS guidelines [19], using the reference values derived by Knudson [20]. Methacholine challenge testing was performed according to a previously published protocol [21]. Normal responsiveness was set at a concentration of methacholine causing a 20% fall (PC_20_) in FEV_1 _of greater than 16 mg*mL^-1^[22].

### Compound Asthma Severity Score

OA severity was calculated according to the Quebec Workers' Compensation Board Scale for OA: 0% = low severity, 100% = maximum severity [23]. This scale assesses three factors in the same way as the one proposed by the American Medical Association [24]: level of bronchial caliber, degree of bronchial responsiveness, and need for medication to control asthma [25].

### Statistical analyses

Continuous data are reported as means ± standard deviations or medians and 25 and 75 percentiles. Proportions were compared by using Chi-Square or Fisher's exact test if the expected cell count was < 5. Continuous variables were compared by using the Mann-Whitney U test. For all data analyses, we used the statistical software package SPSS V.19 (SPSS Inc., Chicago, USA). A p-value of < 0.05 was considered statistically significant.

### Measures of reliability

Cronbach alpha's were calculated to assess the internal consistency of the AQLQ(S) total score and each subscale score. Cronbach's alpha is a numerical coefficient for reliability. The coefficient value ranges from 0 to 1, and the higher the score, the more reliable is the generated scale [26].

### Measures of validity

We calculated Spearman's rho for correlation analyses between two continuous variables, and we conducted point-biserial correlations which allow measurement of the correlation between a continuous variable (AQLQ(S) or PSI total or subscale scores) and a dichotomous variable (*any psychiatric disorder *or *no psychiatric disorder *according Prime-MD) [27]. The correlation was considered small for correlation coefficients between 0.1 and 0.3, medium between 0.3 and 0.5 and high between 0.5 and 1.

### Receiver operator characteristic (ROC) curve and Youden Index (YI)

In order to determine the best cut-off level of the AQLQ(S) emotion subscale score for the diagnosis of clinically relevant anxiety and depressive symptoms according to the PSI, and mood and/or anxiety disorders according to the PRIME-MD, a ROC curve was plotted. The YI was calculated to capture the performance of the diagnostic test and to obtain the cut-off in the AQLQ(S) emotion subscale score. YI was calculated as follows: (Sensitivity+Specificity)-1 [28].

### Linear and logistic regression models

As data of the dependent variable (PSI anxiety and depression subscale scores) were not normally distributed we performed logarithmic transformation and then, applied the second power to this data prior to performing linear regression. The presence of a *psychiatric disorder *(mood and/or anxiety) was assess by the PRIME-MD and coded as a dichotomous variable (yes, no). We used the presence of psychiatric disorder as dependent variable and entered the questions of the AQLQ(S) emotions subscale score as independent variables in the model. We used the automatic stepwise procedure of the statistical package with a probability of F ≤ 0.05 to enter and the probability of F ≥ 0.10 to remove the co-variate.

## Results

### Patient characteristics

Seventy-three subjects were eligible to participate during the study period. We were unable to contact five subjects and eight subjects refused to participate, yielding a final sample of 60 subjects and a participation rate of 82%. Participants did not differ from non-participants with respect to sex, age at diagnosis, atopy, smoking status, lung function, hyper-reactivity to methacholine, proportion of subjects with OA caused by low molecular weight agents, and the number of years at the workplace with symptoms (data not shown).

The mean age of participants was 47.2 ± 11.7 years, 75% (n = 45) of which were male. The median duration of exposure to the causal agent was 10.5 years (Q1;Q3: 3.1;22.8 years). A total of 42% (n = 25) were current smokers. Fifty-five percent (n = 33) of participants were working at the time of re-evaluation, 20% (n = 12) were retired, 20% (n = 12) were unemployed, and 5% (n = 3) were currently on re-training for another job. Thirty-one percent (n = 19) reported their overall health status as being fair or poor.

Asthma-specific quality of life, levels of psychological distress, and the frequency of psychiatric disorders assessed at the re-evaluation are shown in Table 1. Thirty five percent (n = 21) had one or more psychiatric disorder, 19 of whom had a mood disorder, 9 of whom had an anxiety disorder, and 2 of whom had both a mood and anxiety disorder according to the PRIME-MD evaluation.

**Table 1:** Description of Quality of Life, Psychological Distress and Psychiatric Disorders

	**Frequency (n (%))**	**Score (median (Q1;Q3))**
**Juniper AQLQ(S)**		
Symptoms		4.5 (3.5;6.0)
Activity limitations		4.6 (3.3;5.7)
Emotional function		5.0 (3.6;6.6)
Exposure to environmental stimuli		4.8 (3.3;5.8)
Total		4.6 (3.6;5.9)
**SGRQ Asthma Symptom Score (%)**		45 (32;71)
**Psychological distress**	(PSI score > 20)	
Depression	32 (53)	23.0 (10.0;33.0)
Anxiety	36 (60)	24.0 (12.8;39.0)
Anger	30 (50)	19.0 (8.0;33.0)
Cognitive disturbance	31 (52)	24.5 (8.0;39.8)
Total	34 (57)	24.5 (10.8;37.8)
**Prime-MD**		
*Any psychiatric disorder*	21 (35)	
*Any mood disorder*	19 (32)	
- Major depression	8 (13)	
- Dysthymia	3 (5)	
- Minor depression	11 (18)	
*Any anxiety disorder*	9 (15)	
- Panic disorder	1 (2)	
- Generalized anxiety	1 (2)	
- Other anxiety disorder	7 (8)	

Legend: AQLQ(S) = Standardized version of the Juniper Asthma Quality of Life Questionnaire; SGRQ: St. George Respiratory Questionnaire; PSI = Psychiatric Symptom Index, PRIME-MD = Primary Care Evaluation of Mental Disorders questionnaire

### Psychometric properties of the AQLQ(S)

The internal consistency for the AQLQ(S) in this sample was high, with Cronbach alpha's of 0.934 for the emotion, 0.951 for the symptom, 0.922 for the activity, and 0.819 for the environment subscale scores, and 0.936 for the total score. The observed cross-sectional correlations between the AQLQ(S) total and subscale scores with the compound asthma severity score incorporating lung function, bronchial hyperactivity, and current medication support a discriminative validity of the AQLQ(S) and can be seen in Table 2.

**Table 2:** Correlation of the Asthma Quality of Life Questionnaire by Juniper with Psychiatric Symptom Index, the PRIME-MD evaluation, the St.George Respiratory Questionnaire and the Asthma Severity Compound Score

	AQLQ Symptoms	AQLQ Activity limitations	AQLQ Emotional functions	AQLQ Exposure to environmental stimuli	AQLQ Total Score
Asthma Severity Compound Score	-0.582*	-0.611*	-0.627*	-0.561*	-0.650*
					
SGRQ Symptom Score	-.686*	-.650*	-.610*	-.574*	-.693*
					
PSI Anxiety	-.623*	-.648*	-.609*	-.616*	-.664*
PSI Anger	-.331*	-.376*	-.344*	-.404*	-.367*
PSI Depression	-.558*	-.611*	-.507*	-.639*	-.605*
PSI Cognitive disturbance	-.426*	-.401*	-.373*	-.432*	-.419*
PSI Total Score	-.583*	-.613*	-.553*	-.622*	-.619*
					
PRIME-MD Anxiety	0.254	0.163	0.261§	0.213	0.236
PRIME-MD Mood	0.352*	0.381*	0.334*	0.402*	0.393*
PRIME-MD Psychiatric disorder	0.396*	0.361*	0.389*	0.427*	0.417*

Legend: * p < 0.01, § p < 0.05

### Correlation between AQLQ and asthma severity

We investigated the construct validity of the AQLQ(S) and calculated correlation coefficients for the different AQLQ(S) scores with the compound asthma severity score according to the Quebec Workers' Compensation Board Scale for OA as well as the symptom score of the SGRQ. All combinations of scores between the continuous measures yielded significant medium to high correlations (Table 2).

### Correlation between AQLQ and measures of psychological distress

We then calculated correlation coefficients for the different AQLQ(S) scores with the PSI total and subscale scores. All combinations of scores between the continuous measures yielded significant medium to high correlations, which can be seen in Table 2. The highest correlation was found between the PSI anxiety subscale score and the AQLQ(S) total score and most of the AQLQ(S) subscale scores.

**Figure 1:**
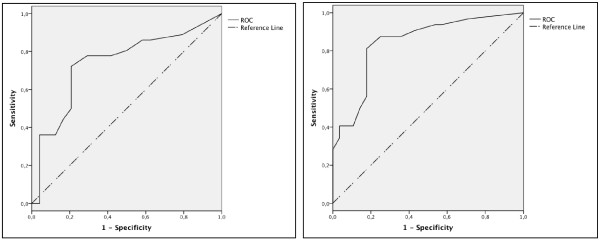
**Receiver operator curves for the AQLQ(S) emotion subscore in the diagnosis of clinically significant anxiety or depression**. ROC = Receiver operator characteristic curve. ROC for AQLQ(S) emotion subscore in the diagnosis of clinically significant anxiety (> 20 points in the PSI anxiety subscore; AUC 0.740 (95%CI 0.608-0.872)). ROC for AQLQ(S) emotion subscore in the diagnosis of clinically significant depression (> 20 points in the PSI depression subscore; AUC 0.843 (95%CI 0.741-0.945)).

**Figure 2:**
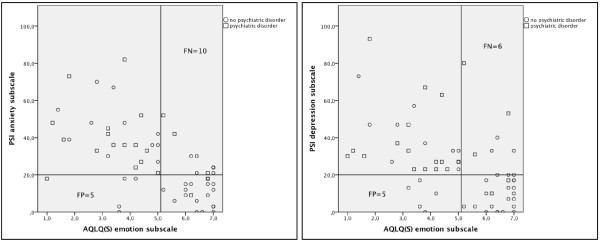
**Scatterplots showing the correlation of the AQLQ(S) emotion with the PSI anxiety subscale and the correlation of the AQLQ(S) emotion with the PSI depression subscale**. PSI = Psychiatric symptom index; AQLQ(S) = Asthma Quality of Life Questionnaire; FP = false-positive; FN = false-negative.

We then investigated the diagnostic properties of the AQLQ(S) emotion subscale for the detection of clinically significant levels of psychological distress according to the anxiety and depression subscales of the PSI. The highest Youden index of 0.53 was obtained when choosing less than 5.1 points as the cut-off for the diagnosis of clinically significant anxiety according to the PSI (Figure 1). Fifteen individuals were misclassified: Ten individuals had AQLQ(S) scores greater than 5.1 points but were classified as having scores greater than 20 points on the PSI anxiety subscale, whereas 5 individuals had scores than 5.1 points on the AQLQ(S) emotion subscale but had scores less than 20 on the PSI anxiety subscale (Figure 2). By using this cut-off sensitivity was 72%, specificity 79%, positive predictive value 84% and negative predictive value 66% for the diagnosis of clinically significant anxiety symptoms according to the PSI. The highest Youden index of 0.63 was obtained when choosing less than 5.1 points as the cut-off for the diagnosis of clinically significant depressive symptoms (Figure 1). Eleven individuals were misclassified: Six individuals had AQLQ(S) scores greater than 5.1 points but were classified as having scores greater than 20 points on the PSI depression subscale, whereas 5 individuals had scores less than 5.1 points in the AQLQ(S) emotion subscale but had scores less than 20 points on the PSI depression subscale (Figure 2). By using this cut-off sensitivity was 81%, specificity 82%, positive predictive value 84% and negative predictive value 79% for the diagnosis of clinically significant depressive symptoms according to the PSI.

When performing linear regression on the PSI anxiety subscale score as the dependent variable, the questions "feeling concerned about having asthma" and "feeling afraid of getting out of breath" were significantly associated (ß = -0.222 S.E. 0.106, p = 0.041 and ß = -0.220, S.E. 0.104, R2 = 0.040). The question "feeling afraid of getting out of breath" was associated with the PSI depression subscale score and the PSI total score (ß = -0.332 S.E. 0.084, p < 0.001, R2 = 0.231 and ß = -0.392, S.E. 0.078, R2 = 0.313, respectively).

### Correlation between AQLQ and measures of psychiatric disorders

We calculated point bi-serial correlations with individuals having an anxiety disorder, mood disorder, or any psychiatric disorder according to the PRIME-MD. Having any mood or any psychiatric disorder showed significant correlations in the medium range for all the AQLQ(S) subscale scores and the AQLQ(S) total score. There was a small point-biserial correlation between having anxiety disorder and the AQLQ(S) emotional function subscale score, but not with the AQLQ(S) total score.

For classifying patients with any psychiatric disorder according to the PRIME-MD, a cut-off of less than 4.7 points in the AQLQ(S) emotion subscale misclassified 17/60 individuals: Six individuals had AQLQ(S) scores of greater or equal 4.7 points but were classified as having at least one psychiatric disorder, whereas 11 individuals had scores less than 4.7 points but were classified as not having a psychiatric disorder according the PRIME-MD (sensitivity 71%, specificity 72%, positive predictive value 58% and negative predictive value 82%, area under the curve 0.736 (95% CI 0.609-0.863; data not illustrated)). When classifying patients with any psychiatric disorder according the PRIME-MD, 4 out of 21 individuals had scores of ≤ 20 points on the PSI anxiety subscale and/or AQLQ(S) scores of ≥ 5.1 on the emotion subscale. Five out of 21 individuals with a psychiatric disorder had scores of ≤ 20 points on the PSI depression subscale and/or had AQLQ(S) scores of ≥ 5.1 on the emotion subscale (Figures 2a and 2b).

After conducting stepwise logistic regression with any anxiety disorder according PRIME-MD as dependent variable, the question "feeling concerned about the need to use medication" was the only one with a marginal association (ß = 0.346, S.E. 0.178, p = 0.052, Cox&Snell R2: 0.062). When any mood disorder and any psychiatric disorder were used as dependent variables, the question "feeling afraid of getting out of breath" showed significant association with both (any mood disorder = ß = 0.456, S.E. 0.165, p = 0.006, Cox&Snell R2: 0.137; any psychiatric disorder = ß = 0.508, S.E. 0.169, p = 0.003, Cox&Snell R2: 0.166).

## Discussion

We found high correlations between impaired asthma-specific quality of life and standard measures of psychological distress, and moderate correlations between impaired asthma-specific quality of life and psychiatric morbidity (i.e., mood and anxiety disorders) in individuals with OA. A cut-off value of < 5.1 on the AQLQ(S)'s emotion subscale could reliably identify individuals with clinically significant levels of depressive and/or anxiety symptoms who need further evaluation by an validated psychiatric interview.

There is limited evidence about the association of psychological stress and asthma morbidity in individuals with OA [29]. When considering the available evidence about the impact of this stress on individuals with non-occupational asthma, we can imagine that an additional psychological burden is associated with OA. This is in accordance with past findings where subjects with OA had slightly but significantly higher impairment in asthma-specific quality of life than those with non-occupational asthma, even when controlling for asthma severity [30]. In a study of asthmatics affiliated with a health maintenance organization in the USA, patients with work exacerbated of asthma had lower quality of life measured according to the mood disturbance, social disruptions, and health concerns subscales of the Mark's Asthma Quality of Life questionnaire, compared to those individuals with no work exacerbated asthma [31].

Various and probably many unknown factors contribute to impairment of quality of life in individuals with asthma. Malo and co-workers have reported a weak but significant correlation between the original AQLQ with FEV1, bronchial responsiveness, and asthma severity in a more extensive sample of individuals with occupational and non-occupational asthma [30]. The AQLQ(S) used in our study and the original AQLQ questionnaires distinguish themselves on one point: in the AQLQ(S)'s five generic activities (strenuous exercise, moderate exercise, work-related activities, social activities, and sleep) replaced specific activities that could be chosen by the patient in the original questionnaire [6]. We could reproduce these findings and could find a larger correlation of the AQLQ(S) subscales and total score with the objective asthma severity score. We also found a large correlation between the symptom domain of a widely used quality of life questionnaire in chronic obstructive lung disease - the St. George Respiratory Questionnaire, which provides support for quality of life being related to factors other than objective markers of disease severity.

It is currently unknown how treatment of psychological distress or psychiatric morbidity (either using psychotherapy or pharmacotherapy) might affect asthma and psychosocial outcomes in individuals with OA. Disease management programs for major depressive disorders have been shown to be beneficial in reducing the severity of the depression, maintaining employment, increasing short term adherence to medication and improving the individuals quality of life while being cost-effective [32]. It a recent systematic review, Lerner and Henke have shown that individuals with depression have higher unemployment rates, more absenteeism and lower at-work performance than individuals without depression [33]. When on medical leave, individuals with poor mental health are at risk for prolonged work absence [34]. Co-morbid psychiatric disorders are one of the reasons for the adverse socioeconomic outcomes in regards of unemployment and income loss. As these disorders can influence the individual's adherence to medication, lifestyle behaviors such as smoking and managing environmental asthma triggers, it could at least partially explain the persistent symptomatology and bronchial hyperresponsiveness in many individuals with OA seen even years after termination of the exposure to a sensitizing agent [35,36].

We found medium to large correlations between the individual AQLQ(S) and the PSI. In point-biserial correlations between AQLQ(S) and PRIME-MD outcomes such as having psychiatric disorder the correlations were in the range small to medium. In a population sample in Australia, major depression according to the PRIME-MD was associated with dyspnoea, wakening at night and morning symptoms in asthmatics and these symptoms were shown to have the greatest impact on decrease in quality of life scores in the SF-36 [37]. When using the Hospital Anxiety and Depression (HAD) scale, Rimington and co-workers found moderate correlation of the HAD depression subscale and a somewhat lower correlation of the HAD anxiety subscale with the AQLQ symptoms subscore in a sample of asthmatic patients attending GP offices in the UK [38]. In that study, hardly any correlation of HAD anxiety and depression subscales on the one hand and lung function expressed as forced expiratory volume in one second (FEV1) or peak flow on the other hand could be demonstrated [38]. In contrast, Hommel and co-workers reported anxiety and depression to influence asthma specific quality of life measured with the Living with Asthma Questionnaire (LWAQ). When performing regression analysis, they demonstrated that anxiety had an independent main effect on LWAQ when the model was controlled for depression [39]. The impact of concomitant depression and anxiety seems to be even more deleterious for health related quality in life in individuals with chronic obstructive pulmonary disease [40].

The AQLQ(S) has been used in a large variety of clinical therapeutic trials and many cross sectional studies on patients with OA. Measuring quality of life with the AQLQ(S) allows to determine the impact of asthma on respiratory symptoms, emotional function, activity limitation as well as environmental stimuli. These factors are important to acknowledge in clinical practice when assessing a patient with asthma. In our sample of individuals with OA who have been removed from exposure to the sensitizing agent, using a cut-off point of 5.1 in the emotional function subscale most reliably distinguishes individuals with significant psychological distress, whereas a cut-off of 4.7 can be used to identify individuals who are at risk of relevant psychiatric disorder according to the PRIME-MD evaluation. It is not the intention of the authors to suggest that the evaluation of patients with the AQLQ(S) emotional subscale can replace a structured diagnostic interview by a psychiatrist - which is considered to be the gold standard - for the diagnosis of psychiatric diseases. Questionnaires such as the Hospital Anxiety and Depression Scale (HADS) have been shown to have a sensitivity of 66-78% and specificity of 83-97% for the diagnosis of either depression or anxiety disorders in a general practice setting [41]. Therefore even administration of tools specifically designed to screen for psychiatric disorders would not allow making an accurate diagnosis and starting treatment without performing a structured psychiatric interview. The advantage of using the AQLQ(S) questionnaire is that it is widely available and regularly used in clinical practice and trials. It can therefore be used as a screening test. Considering the results of the emotional subscale will not only allow to measure the impact of asthma on quality of life but also to identify some individuals in whom a more extensive investigation such as a structured psychiatric interview is warranted. The diagnostic performance of the test using a cut-off of < 5.1 in the emotional subscale score of the AQLQ(S) is modest for identification of clinically important psychological distress according to the PSI. But the performance is less for the diagnosis of current psychiatric disorders according to the PRIME-MD which relies on the diagnostic criteria for depressive and anxiety disorders according to the Diagnostic and Statistical Manual of Mental Disorders, 4^th ^Edition, DSM-IV. In fact the positive predictive value for the diagnosis of current psychiatric disease by using a cut-off of 4.7 is close to the predictive value of flipping a coin and would even be lower when this test would be performed in a population with a lower prevalence of psychiatric disorders. There is very limited data suggesting that anxiety and depression are more common in workers in whom the asthma is related to the workplace [42]. In our study all individuals were compensated for OA and one could expect that the prevalence of mood and anxiety disorders is more prevalent in this population than in most other populations of asthmatics. However there is currently no data available to conclude that the prevalence of psychiatric disorders is lower in individuals with uncompensated OA or in individuals with work-exacerbated asthma. Further studies are needed to compare the correlation of psychiatric disorders and psychological distress with asthma specific quality of life measures, such as the AQLQ(S), in individuals with OA, work-exacerbated asthma and asthma that is unrelated to the workplace [41].

The AQLQ(S) emotional function subscale respondents report on different aspects that have been grouped in this domain by the developers of this questionnaire in which the items relate to three broad dimensions (concerns, anger and anxiety). When considering individual questions of this domain, the one about feeling afraid of getting out of breath was significantly associated with the PSI depression subdomain and PRIME-MD mood disorder whereas the questions about feeling concern about having asthma and about the need to use medication were significantly associated with PSI anxiety levels and PRIME-MD anxiety disorders respectively. Since our analysis was descriptive, we do not suggest to reduce items that have not shown significant correlation with psychologic distress or psychiatric disorders from the emotions subdomain of the AQLQ(S) questionnaire.

Whereas the PSI is a continuous measure that provides information about the number and severity of psychological symptoms, PRIME-MD diagnoses are categorical: individuals are classified as having a particular disorder or not based on having fulfilled a defined number of diagnostic criteria. Therefore, the severity of psychiatric disorders as measured by the PRIME-MD cannot be quantified [43]. Clinically, anxiety and depressive symptoms (and disorders) overlap significantly, so it can sometimes be difficult to determine if these disorders are separate entities or different manifestations of the same disorder [39]. Due to the limited number of individuals with OA included in our study, we were not able to examine associations between AQLQ(S) scores and individual mood and anxiety disorders (e.g. panic disorder, generalized anxiety disorder). To demonstrate these associations, studies with larger samples of individuals with OA are needed. Further our sample consisted of manly male workers with OA and therefore one must be cautious when extrapolating our findings to a population of female workers as the prevalence of the different forms of psychiatric disorders might be different [9].

Our study does not allow us to determine the relation of causation. We did not have information available about psychological distress or any psychiatric disorder prior to the development of OA or at the time the diagnosis of OA was made. Furthermore, we did not gather information about concurrent or past behavioural or medical therapy for psychiatric disorders in each individual. To our knowledge, the prevalence of psychiatric disorders at the time of diagnosis of OA and its devolution after removal from the causing agent or workplace is currently unknown and thus other studies are needed to investigate these factors in prospective investigations. It is unclear how interventions specifically targeted to decrease psychological distress or psychiatric disorders change the natural course of both conditions OA and concurrent mental disorders.

An important strength of the present study is that extensive objective assessments including spirometry, measurement of nonspecific bronchial hyperreactivity, and specific inhalation testing were performed in all individuals, the latter of which is considered the reference standard for a diagnosis of OA [44,45].

## Conclusions

Our study suggests that it is important to consider concomitant psychological distress and psychiatric morbidity in individuals with OA, even when their exposure to the causing allergen has ended. By performing disease-specific quality of life assessment with the AQLQ(S), individuals with significant psychological distress or psychiatric disorder could be identified and more elaborative and conclusive investigations and if necessary treatment be offered.

## Abbreviations

AQLQ(S): Juniper Asthma Quality of Life Questionnaire; CSST: "Commission de la santé et sécurité du travail du Québec" translates as Workers' Compensation Agency of Quebec; FEV1: forced expiratory volume in one second; FN: false-negative; FP: false-positive; HAD: Hospital Anxiety and Depression Scale; OA: Occupational Asthma; LWAQ: Living with Asthma Questionnaire; PC_20: _Concentration of Methacholine causing a 20% fall in FEV_1; _PRIME-MD: Primary Care Evaluation of Mental Disorders; PSI: Psychiatric Symptom Index; ROC: Receiver Operator Characteristic Curve; SGRQ: St-Georges Respiratory Questionnaire; YI: Youden Index.

## Competing interests

The authors declare that they have no competing interests.

## Authors' contributions

DM and JLM won funding for this project. DM, KLL, HG and JLM designed the study. JA recruited participants and conducted interviews and managed the database. DM analyzed the data and wrote the first draft of the manuscript. KLL, HG and JLM provided support in overseeing the data analysis and revision of the drafts. All authors commented on and contributed to this manuscript. DM is the guarantor for this manuscript. All authors read and approved the final manuscript.

## Funding

Center for Asthma in the Workplace, Centre Léa-Robback sur les inégalités sociales de la santé, Canadian Institutes of Health Research. David Miedinger is the recipient of a research grant from the Swiss National Science Foundation (PBBSB-120767) and from the Center for Asthma in the Workplace (Canadian Institutes of Health Research). Kim Lavoie is supported by a salary award from the Fonds de la recherche en santé du Québec (FRSQ).
